# HEALTHY AGEING FOR ALL? SOCIOECONOMIC CHANGES OVER TIME IN DISABILITY-FREE AND INDEPENDENT LIFE EXPECTANCIES

**DOI:** 10.1093/geroni/igz038.3295

**Published:** 2019-11-08

**Authors:** Holly Q Bennett, Fiona E Matthews, Louise Robinson, Lynne Corner, A Kingston, Carol Jagger

**Affiliations:** Newcastle University, Newcastle upon Tyne, United Kingdom

## Abstract

Background: Previous research using cross-sectional data shows widening inequalities in disability-free life expectancy (DFLE) by socioeconomic status (SES) in the UK. We aimed to understand the underlying transitions of DFLE and years dependency free (DepFLE) using longitudinal data from the Cognitive Function and Ageing Studies (CFAS I and CFAS II). Methods: Two large population based studies of those aged 65+ in three centres (Newcastle, Nottingham, Cambridgeshire) interviewed at baseline in 1991 (CFAS I) and 2011 (CFAS II) with follow up two years later. Disability was measured using difficulty in activities of daily living (ADL), and dependency by time between help required for ADLs. SES was based on area deprivation categorised into study specific tertiles. Transitions between disability or dependency states and death were modelled using Interpolated Markov Chain software. Results: Between 1991 and 2011, DFLE and disabled life expectancy (DLE) at age 65 increased for men in every SES group, with men being less likely to become disabled or die, and more likely to recover, in 2011 than 1991 across SES groups. For the most disadvantaged women, DFLE was similar, and DLE increased, whilst for the remaining women DFLE increased and DLE was similar. For women probability of recovery increased and probability of death from disability decreased but probability of becoming disabled decreased only for the most advantaged. DepFLE patterns across time were similar but more pronounced. Conclusion: Preventive measures should focus on reducing the disability and dependency onset in the most disadvantaged to ensure inequalities do not widen further.

